# Composting of organic fraction of municipal solid waste in a three-stage biodegradable composter

**DOI:** 10.1016/j.heliyon.2024.e37444

**Published:** 2024-09-04

**Authors:** Dakshesh Chimanbhai Saypariya, Deval Singh, Anil Kumar Dikshit, Mohan B. Dangi

**Affiliations:** a Environmental Science & Engineering Department, Indian Institute of Technology Bombay (IITB), Powai, Mumbai, 400 076, Maharashtra, India; b Department of Geography and City & Regional Planning, California State University, Fresno, CA, 93740, USA

**Keywords:** Biodegradable organic waste, Germination index, Moisture content, Municipal solid waste, Physical-chemical characteristic, Vertical drum composter

## Abstract

The increase in municipal solid waste (MSW) generation rate has been a growing concern for the modern-day era. On-site composting has been the promising clean-tech alternative to managing biodegradable organic waste (BOW) in MSW. It allows sustainable and compact solutions for the in-house treatment of MSW, reducing the overall burden on landfill and treatment facilities. In this manuscript, a batch and pilot scale performance assessment study were conducted for BOW using a three-stage vertical drum composter (R1, R2, R3). The study aims to determine the impact of aeration, turning mechanisms, bulking agents, degradation rate, and process parameters on compost quality. It was found that physical-chemical properties such as bulk density (0.3 g/cm^3^), pH (∼7), temperature (<50 °C), moisture content (<20 %), total volatile solids (33 %), electrical conductivity (<4 dS/m) and carbon/nitrogen ratio (∼16) of final compost was under the prescribed limit. We conclude that the provision for aeration via perforated vents and regular turning mechanisms substantially impacted the quality of compost. Compost maturity was determined using humic to fulvic acid (HA/FA) ratio and germination index (GI). The HA/FA and GI of final compost in R1, R2, and R3 were found to be 6.21, 7.22, and 6.90; and 85.3 %, 90.4 %, and 87.6 %, respectively. During the degradation process, the increasing trend of HA/FA ratio (5–8) and GI (>85 %) showed that the compost quality was rich in nutrients and soil-conditioning properties. Based on the kinetic study, it was conclusive that adding bulking agents in R3 (0.0078 day^−1^) and R4 (0.0098 day^−1^) contributed to high degradation rates, underlining the value of creating a porous structure that enhances microbial activity. The findings can be a resource for waste generators, managers, technocrats, and policymakers to tackle the issues related to in-house management and treatment of MSW.

## Highlights

• Pilot-scale assessment of three stage vertical drum composter for 45 days.
• Investigating the impact of bulking agent, aeration and mixing on compost quality.
• Germination index and HA/FA ratio was >85 % and 13.5–14.5 in compost samples.
• Compost quality was rich in nutrients and soil-conditioning properties.

## Graphical abstract

**Figure:**
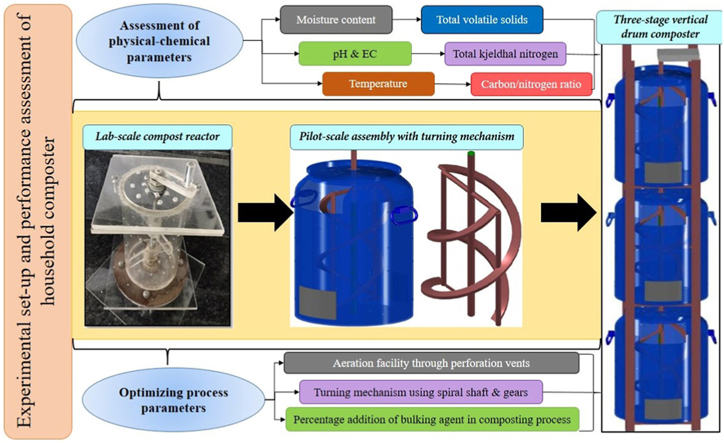

**Table:** Abbreviation

BA	Bulking agent
BOW	Biodegradable organic waste
C/N	Carbon to nitrogen ratio
Cu	Copper
EC	Electrical conductivity
FA	Fulvic acid
GI	Germination index
GW	Garden waste
HA	Humic acid
HA/FA	Humic to fulvic acid
HP	Horsepower
HCl	Hydrochloric acid
HNO_3_	Nitric acid
ICP-AES	Inductively coupled plasma atomic emission spectroscopy
IW	Inorganic waste
k	Degradation rate constant (day^−1^)
MC	Moisture content
MSW	Municipal solid waste
NaOH	Sodium hydroxide
Ni	Nickel
Pb	Lead
rpm	Rotation per minute
TKN	Total Kjeldahl Nitrogen
TVS	Total volatile solids
ULBs	Urban local bodies
VDC	Vertical drum composter
Zn	Zinc

## 1 Introduction

The increase in municipal solid waste (MSW) generation and its lack of proper management has been a global concern for both developed and developing nations [[1], [2], [3]]. This poses a significant challenge to managers (urban local bodies (ULBs)) and producers (residents) to counter the issues related to MSW collection, treatment, and disposal [1,4]. It also significantly impacts community health and the environment, leading to adverse effects such as air pollution and soil and groundwater contamination [[5], [6], [7]]. In 2016, the estimated global MSW generation was 2.01 billion metric tons (mt)/year, expected to surpass 3.4 billion mt/year by 2050 [8,9]. A staggering 1.3 billion mt of MSW is generated annually, equivalent to an average daily rate of 1.2 kg per capita [[8], [9], [10]]. Projections indicate that the MSW quantity is expected to surge to 2200 mt/year by 2025 [8,9]. Generally, within MSW composition, biodegradable organic waste (BOW) (50%–60 %) consists of kitchen waste, raw vegetables, fruit peels, and cooked food waste [[11], [12], [13], [14], [15]]. The reuse, recycling, and recovery of value-added products from the BOW have been a growing topic for most of the developing nations [9], particularly the South and Southeast Asian countries (India, Bangladesh, China, Nepal, Sri Lanka, etc.) comprising of minimum on-site and off-site treatment facilities [4,9,16]. Also, composting, anaerobic digestion, incineration, recycling, and landfilling are the most commonly preferred methods for MSW treatment and disposal [1,17].

Composting and anaerobic digestion are frequently used biological conversion methods for BOW within MSW [1]. Composting has become an attractive alternative for stabilizing organic waste due to its simple on-site implementation [1,17]. It involves aerobic biodegradation and stabilization of organic waste under-regulated, thermophilic, and aerobic conditions into compost [1,18,19]. At a decentralized level, household composting presents a promising solution that empowers individuals to manage their organic waste locally [20,21]. Household-level composting alleviates the strain on centralized waste management systems, which reduces transportation costs and facilitates recycling organic waste into a valuable resource [22,23]. However, the effectiveness of composting systems hinges on various critical factors, including physical-chemical properties, ambient conditions, turning frequency, and bulking agents [23]. These factors influence the rate of degradation, compost quality, and the overall efficacy of the composting process.

In the past few years, most of the studies have focused on developing centralized facilities for composting (windrow and pit composting systems) for more extensive groups of settlements [[20], [21], [22], [23]]. On the contrary, the decentralized compost systems (in-vessel composter, rotary drums, etc.) are much more compatible with households, as they require less space, low cost, and energy consumption. Presently, limited studies have investigated different in-house prototypes and methods for composting [[20], [21], [22], [23]]. The aim was to examine the factors that influence the process and the quality of the compost. Therefore, a group of researchers conducted a study to investigate the impact of different aeration techniques in the composting process, such as forced aeration static pile (FAS), pile turning (PT), natural ventilation static pile (NVS), and combined process (PT + NVS) [24]. The NVS system initially showed higher temperatures (>30 °C) and maintained a final temperature of 10 °C. Comparing NO_3_^−^N content, C/N ratio, CO_2_ impact, and maturity, the combined process required more energy but outperformed all other techniques [24]. Similarly, other researchers conducted studies to evaluate the performance efficacy of in-vessel composting under varying operating conditions with different organic substrates [25,26,27]. The findings from the above studies can be summarized as: (a) the mechanized reactor was proved to be more efficient than the manual reactor since it had a 10–15 % higher volume reduction rate, (b) a significant percentage reduction of 20–25 % was found in total organic carbon to volatile solid ratio, and (c) addition of bulking agents (wood chips and yard waste) enhanced the quality of compost and reduced the retention time phase.

Although there is substantial literature on in-house composting systems, there is a lack of comprehensive performance assessments for household composters specifically designed for BOW within MSW [17,[25], [28], [29], [30]]. Therefore, our study hopes to propose an optimized operational standard for aeration rate, turning frequency, type of bulking agents, and other process parameters (pH, temperature, moisture content, organic content, etc.), which are essential for composting. The present study aims to investigate the following objectives.

(a) To understand the impact of aeration and turning mechanisms on compost quality.
(b) To anticipate the influence of bulking agents on leachate control and odor management.
(c) To investigate the role of process parameters and their influence on the quality of the final compost as it can accelerate the practical application of household composting.
(d) To determine the compost maturity and germination test as it provides practical insights into the nutrient-rich and soil-conditioning properties of the compost.

Besides the above-outlined objectives, the study also encompasses certain limitations, as mentioned below (by addressing these limitations, we aim to understand the research scope and identify future research areas comprehensively).

(a) The study focused on the BOW of MSW. Separate studies might be necessary for other types of waste or mixed waste streams to understand the impact of operational parameters on compost quality.
(b) The findings are based on specific climatic conditions that may influence the composting process. These results might not apply to regions with different climates.
(c) The study did not address the economic feasibility of the composting process, including costs for equipment, energy consumption, and labor for aeration and turning. These factors are crucial for practical implementation.
(d) The study primarily examined compost quality and process efficiency but did not extensively evaluate environmental impacts such as greenhouse gas emissions or leachate production during composting.

## 2 Materials and methods

### 2.1 Sample collection and preparation

The sample size of 50 residential apartments was selected for the MSW study. The selected apartments were situated inside the Indian Institute of Technology Bombay (IITB) campus in Mumbai, India. A day before sampling, a notice was circulated among residents instructing source segregation of MSW into two major categories: (a) BOW comprising food and kitchen waste, and (b) inorganic waste (IW) comprising plastic, paper, cardboard, glass, metals, etc. During the sample collection process, the segregated waste was checked and placed in two containers designated for BOW and IW. Besides this, garden waste (GW) (dried leaves, wood chips, and other plant-based residues) was also collected from nearby parks located within the campus area. The GW was used as a bulking agent with BOW in a batch-scale study. The BOW to GW ratio used for the study was 4:1. Generally, the bulking agent provides structure strength, improves airflow, and prevents compaction, facilitating the aeration and decomposition of organic matter. Finally, 28 kg of BOW was collected manually using laborers and field operators, primarily comprised of fruits and vegetable peels, leftover cooked food, egg cells, chicken bones, fish bones, etc. The IW consisted of plastic bags, tea bags, packaging materials, hair, bandages, papers, cardboard products, etc. Later, the cone and quarter method was used to classify two sets of BOW sample groups, i.e., 10 kg and 18 kg for batch and pilot scale studies, respectively [4]. Also, the particle size reduction was performed using shredder equipment for both sets of BOWs.

### 2.2 Batch scale study

The batch scale study was carried out using four small-size compost reactors (R1, R2, R3, and R4). The aim was to validate the degradation rate and variation in process parameters such as temperature, moisture content (MC), total volatile solids (TVS), pH, electrical conductivity (EC), and carbon/nitrogen (C/N) ratio. Each set of reactors was simulated under different combinations and physical conditions (Table 1). The present study investigated the impact of four major operating parameters: (a) Peripheral perforations – It improves aeration, promoting aerobic microbial activity and resulting in uniform, nutrient-rich compost; (b) Turning mechanism – It ensures even distribution of moisture and temperature, preventing compaction and enhancing decomposition efficiency, (c) Addition of bulking agent – It increases porosity and airflow, balancing moisture and accelerating decomposition for high-quality compost. The detailed operation parameters for reactors R1, R2, R3, and R4 have been discussed in Table 1. Reactor R4 served as the control, incorporating all the operating conditions. The individual reactor had a holding capacity of 1.5–1.7 L, with an inner diameter of 12 cm and a length of 15 cm. The reactors were fabricated using an acrylic sheet (10 mm thick) and acrylic pipe, as shown in Fig. S1. A manual turning mechanism made up of a stainless-steel shaft along with acrylic blades was incorporated in R2 and R4 to understand the impact of regular mixing on compost quality. Fig. S1(a) represents a detailed diagrammatic understanding of the turning mechanism used in reactors. Besides this, peripheral holes (3 mm size with 2.5 cm c/c spacing) were provided along the cylindrical surface of reactors to ensure the passive diffusion of air into the reactors. A leachate collection base was provided in all the reactors, as shown in Fig. S1(b).

**Table 1:** Different operating conditions for lab-scale reactors R1, R2, R3 and R4.

Activities	Batch scale reactors			
	R1	R2	R3	R4
Peripheral perforations	✓	✓	✓	✓
Turning	×	✓	×	✓
Addition of bulking agent	×	×	✓	✓

### 2.3 Kinetic study

Kinetic study is vital in providing insights related to organic decomposition rates, which helps in process optimization. This information aids in managing composting timeframes efficiently, reducing environmental impact, and producing high-quality compost for soil improvement, agriculture, and waste diversion. It also provides insight into the rate of biodegradables consumed by microorganisms during the process. The degradation of organic matter as a function of time follows first-order kinetics as discussed in (1), (2) [31].

The degradation rate constant was obtained by plotting ln(C/Co) versus time (t) for all four reactors. After determining the degradation rate, reactor R4 had a relatively higher degradation rate than the other three reactors. Hence, in designing a three-stage vertical drum composter, conditions of reactor R4 were considered and devised accordingly.

(1) $$\frac{d\left(C\right)}{dt}=-k∙C$$

After integrating Eq. (1) with the initial condition as C = C_o_ when t = 0, gives

(2) $$\ln\frac{C}{C_{o}}=-k∙t$$

where,

C is the organic matter at any time, k is the degradation rate constant (day^−1^), and t is the time of composting (days).

### 2.4 Pilot scale study

In this research, a pilot-scale study was proposed using a three-stage vertical drum composter (VDC) to overcome the issues related to waste storage, household treatment, and reuse of BOW. It allows efficient and controlled organic waste composting, permitting sequential processing of different stages (filling, maturing, and emptying) to produce high-quality compost with reduced odor and faster decomposition. The findings from the batch scale study were considered and incorporated into the design of VDC. The three-stage VDC's detailed structural layout and capacity building are discussed below.

#### 2.4.1 Structural design of three-stage vertical drum composter

**Fig. 1:**
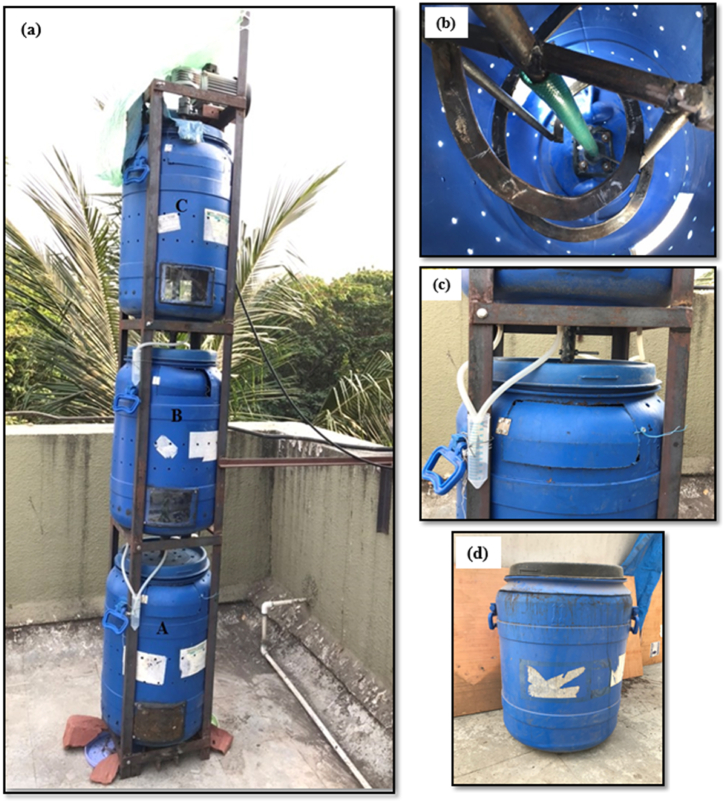
Field-scale deployment of three-stage vertical drum composter prototype: (a) complete structural design and assembly of drums A, B, and C; (b) mechanical turning of BOW using a spiral shaft; (c) provision for leachate collection through the bottom vent of each drum; and (d) drum capacity of 70 L.

The VDC system was made up of three identical plastic drums with a capacity of 70 L each. The drum is labelled as A for the bottom, B for the middle, and C for the top. The detailed 3D design model of the three-stage VDC has been presented in Fig. S2. These three drums were placed vertically on one another and connected with a common central shaft, as shown in Fig. 1(a–d). The complete three-stage VDC had a total height of 230 cm (7.7 feet) with motor assembly. The shaft was connected by coupling it with a simple nut bolt mechanism as shown in Fig. 1(b). The mild steel frame with the bottom platforms was designed and fabricated to support VDC so that one side of the frame could be removed. An electric gear motor of 0.5 HP of 30 rpm was mounted on the top of the shaft enabling the rotation of a typical central shaft as shown in Fig. 1(b). The turning mechanism was attached to the central shaft in each drum. Two spiral plates were used for the turning mechanism in such a way that one plate covers the outer part of the drum, and another covers the inner part of the drum for turning the waste materials in the drums. The spiral plates were used to mix BOW appropriately in horizontal and vertical directions.

A flexible PVC pipe (slightly higher in diameter than the drum's central shaft) was attached to all the drums to cover the central shaft, which helped protect the interference of BOW with the rotating shaft. Each drum had three openings, i.e., two at the bottom and one at the top for sample collection and feeding. The bottom windows were covered with an acrylic sheet during composting. The acrylic sheet cover was removed after the composting cycle to collect and replace the waste for the next cycle. Besides this, multiple perforation vents were provided on the periphery of each drum and the top cover of the drums for passive air diffusion, ensuring the complete system maintained aerobic condition. A spacing of 10 cm was provided between two drums for leachate collection. The provision for leachate collection was made through the bottom nozzles attached below the drums. For Drum A, the leachate was collected in the bottom tray. Meanwhile, for Drum B and Drum C, leachate was collected in a centrifuge tube tied to the steel frame as shown in Fig. 1(c). The detailed dimensions and configurations have been discussed in Table 2.

**Table 2:** Design **s**pecifications of three-stage vertical drum composter.

Drum Label		A, B, C	Control
Drum height (cm)		60	60
Drum diameter (cm)		40	40
Peripheral hole diameter (mm)		7.5	NP^a^
Center-to-center spacing between holes (cm)		5	NP
Top window	Height (cm)	10	NP
	Width (cm)	20	NP
Bottom window	Height (cm)	10	NP
	Width (cm)	15	NP
Spiral plate	Width (cm)	2.5	NP
	Thickness (mm)	4	NP
Central shaft diameter (mm)		20	NP

a NP = Not Provided.

#### 2.4.2 Working methodology

At an interval of every 15 days, the VDC reactors were fed with 1 kg of pre-processed BOW. The reactors A, B, and C were fed in a series and at an alternative period of 15 days, i.e., BOW was fed in reactors A, B, and C at 0, 15, and 30 days. The next cycle of BOW was fed in reactors A, B, and C at a time interval of 15, 30, and 45 days. Therefore, the first set of compost will be prepared on the 15th day from Reactor A. Simultaneously, the continuous turning process was done daily using a centrally attached shaft and electric gear motor at 30 rpm in the clockwise and counter-clockwise direction for around 3–4 min.

The experimental set was performed to understand the effect of bulking agents on compost quality. A varying proportion of bulking agents and BOW was added to all three reactors, as shown in Table 3. The initial stabilization phase of the 15-day cycle is called the degradation phase in all drums. The complete cycle duration for each drum was set to be 45 days. The materials were turned daily once in the clockwise and counter-clockwise direction for around 3–4 min. Temperature variation was measured every day in all the drums. The samples were collected from each set of reactors on every fifth day from the first day of feeding to the end of the cycle, i.e., on the 45th day.

**Table 3:** Percentage of bulking agent addition in drums A, B, C, and control.

Drum Label	A	B	C	Control
% of bulking agent (on a wet basis)	5	10	15	0
Turning	Yes	Yes	Yes	No

### 2.5 Analytical methods

#### 2.5.1 Bulk density

The bulk density of segregated BOW from MSW was determined as per ASTM E1109-18 test protocols [32]. Initially, the BOW was filled in a cylindrical container (1 L) to the top without compaction. The container was lifted and dropped (three times) from 6 cm height using a temporary mechanical arrangement. This allows uniformity and accuracy in the compaction rate for sample sizes used in cylindrical containers. Further, the leftover space at the top of the container was filled with BOW. Finally, the initial and final weight of the cylindrical container was compared to determine bulk density.

#### 2.5.2 Moisture content and total volatile solids

As per the gravimetric method (IS 10158:1982), the MC and TVS were determined for batch and pilot scale composters at 0, 5, 10, 15, 20, 25, and 30 days. The collected sample was pre-weighted and oven-dried at 105 °C for 24 h in a hot air oven. Later, the oven-dried sample was allowed to cool in a desiccator. The initial and final sample weight was analyzed to determine MC. Also, the oven-dried sample was placed in a muffle furnace for 2 h to determine TVS.

#### 2.5.3 Carbon/nitrogen ratio

The C/N ratio for BOW from batch and pilot composter was determined using a ThermoFisher CHNS analyzer (model: Flash smart V CHNS/O) [4]. The adopted instrumental method to determine carbon (C), nitrogen (N), hydrogen (H), and oxygen (O) was as per the British/European standard CEN/TS 15104:2005 [4]. Finally, the estimated value of C and N was used to compute the C/N ratio.

#### 2.5.4 Temperature, pH, and electrical conductivity

The temperature inside the batch and pilot composter was measured daily between 10 a.m. and 11 a.m. using a battery-operated digital temperature probe (DigiTemp 9100). To determine the pH and EC, 50 g of samples were collected from the composter every 5th day. The collected sample was oven-dried and mixed with distilled water in a ratio of 1:10 (w/v). The prepared sample combination was mixed using a magnetic stirrer for 5 min, and solid fractions were allowed to settle. Later, the filtration was performed to extract liquid solvent. Finally, the pH and EC for liquid solvent were determined using a pH meter (Eutech pH tutor) and conductivity meter (Systronic conductivity meter 306), respectively [33].

#### 2.5.5 Heavy metal

The trace elements (copper (Cu), nickel (Ni), lead (Pb), and zinc (Zn)) were determined using acid digestion and inductively coupled plasma atomic emission spectroscopy (ICP-AES) analyzer. The collected fine powdered compost samples (0.1 g) were digested using the solutions of nitric acid (HNO_3_) and hydrochloric acid (HCl) with a ratio of 4:1 (v/v), respectively. The prepared solution was added to Teflon tubes and was digested at 200 °C, where ramp and hold time were 20 min each. The sample tubes were allowed to cool down at room temperature. Later, the solution was transferred to a sampling tube, and a simple two-step microwave-assisted digestion process was followed (**EPA 3051A**) [34]. The digested samples were used in ICP-AES to analyze macro and trash elements.

#### 2.5.6 Total Kjeldahl nitrogen

Total Kjeldahl Nitrogen (TKN) content was measured using the digestion method and TKN analyzer (Tulin equipment) [35]. The dry sample (0.1 g), catalyst mixture (3 g) (1:5 ratio of Copper Sulphate (CuSO_4_) and Potassium Sulphate (K_2_SO_4_)), and concentrated sulphuric acid (H_2_SO_4_) (10 ml) were mixed in digestion tubes. Further, the prepared samples were digested until the color turned bluish-green. After the samples were cooled down, the digested samples were loaded in a 25 ml conical flask with 4 % boric acid and four drops of indicator (Methyl red & Bromocresol green). This prepared sample was kept in a distillation unit to collect the steam and titrated using 0.1N of H_2_SO_4_ until its color changed to pink. Finally, the TKN was measured using Eq. (3).

(3) $$TKN\;\left(\%\right)=\frac{14\times N_{a}\times A_{TV}\times 100}{W\times 1000}$$

where,

N_a_ is the normality of acid, A_TV_ is the actual titrant value, and W is the sample weight.

### 2.6 Maturity test

The maturity of compost samples was determined based on the concentration of humic acid (HA) and fulvic acid (FA). The HA and FA are vital indicators as they reflect the decomposition rate of organic fractions in compost samples [36]. The higher concentration levels signify advanced decomposition rate and stability in compost samples, making them suitable for vegetation. In the present study, the chemical extraction process using Sodium Pyrophosphate Decahydrate (Na4P_2_O_7_⋅10H_2_O) and Sodium Hydroxide (NaOH) was performed to determine the concentration of HA and FA [37]. The mix proportion of 0.1 M of Na_4_P_2_O_7_⋅10H_2_O and 0.1 N of NaOH was prepared with a ratio of 1:1. The dried compost samples were mixed with the prepared proportion in the ratio of 1:10 (w/v, dry weight basis). The prepared combination was kept in an orbital shaker at around 200 rpm for 24 h. Later, the liquid mixtures were centrifuged at 10,000 rpm for 15 min.

Further, the pH of the supernatants was adjusted to 1 with 3 M of HCl. The supernatants were allowed to settle overnight for layer separation at room temperature. After the layer separation, the supernatants were again centrifuged at 10,000 rpm for 15 min. Finally, the supernatants were analyzed to determine the concentration of HA and FA in compost samples using UV–Vis Spectrophotometry (Agilent Technologies, Model: Carry 100 UV–Vis) [38].

### 2.7 Germination test

Generally, the germination test is often referred to as a phytotoxicity test, which is performed to check the quality and assure the applicability of compost in agricultural and other vegetation/plantation activities. This test assesses the impact of compost on plant seed germination and early seedling growth. In the present study, the phytotoxicity test of the compost samples was analyzed based on the germination index (GI) of fenugreek seeds (Trigonella foenum-graecum) as shown in Eq. (4) [28]. The dried powdered sample was mixed with distilled water at 1:10 (w/v). The mixture was centrifuged to maintain the homogeneity in the liquid supernatant left out at the top. Later, the liquid supernatant was separated and filtered from the mixture. Further, the ten fenugreek seeds were placed on the filter paper which was soaked with 5 mL of extracted liquid supernatant. The filter paper and seeds were placed in a Petri dish at 26–28 °C for four days in a dark place. Finally, the root length of all germinated seeds was measured. Besides this, the test was repeated with distilled water as a control.

(4) $$GI\;\left(\%\right)=\frac{{GS}_{com}\times {RL}_{com}\;}{{GS}_{co}\times {RL}_{co}}\times 100$$

where,

GS_com_ and GS_co_ are the numbers of germinated seeds from the liquid supernatant of the compost sample and control, respectively. RLcom and RLcon are the total root length from the liquid supernatant of the compost sample and control, respectively.

### 2.8 Statistical analysis

The mean values and standard deviations of three replicates for each treatment are reported. Data for lab and pilot-scale study was analyzed using a one-way analysis of variance (ANOVA). The least significant difference test was employed to determine significant differences between mean values of different physical-chemical parameters. All statistical analyses were conducted using Microsoft Excel on Windows 2019 (IIT Bombay, India). The statistical hypothesis for the test was assumed as (a) null hypothesis: all the assessed physical-chemical parameters had no significant differences; (b) alternative hypothesis: all the assessed physical-chemical parameters for the above-discussed systems had significant differences.

## 3 Results and discussion

### 3.1 Waste characterization

The physical-chemical parameters for organic waste fractions, i.e., BOW and GW were analyzed using their respective test protocols. The detailed characteristics of BOW and GW have been provided in Table 4. The bulk density, MC, TVS, pH, EC, and C/N ratio of BOW was 513 kg/m^3^, 79.5 %, 90.7 %, 5.6, 2.95 dS/m, and 18.36, respectively. Similarly, the bulk density, MC, TVS, pH, EC, and C/N ratio for GW were 59 kg/m^3^, 19.2 %, 81.1 %, 5.3, 1.4 dS/m, and 26.04, respectively. We also noticed that the higher MC and TVS content in BOW was due to raw vegetables/fruits, half-cooked organic residues, and liquid slurry. However, the MC in GW was less than BOW, primarily due to dried leaves, plant residues, and other lignocellulose biomass. Studies have also reported higher MC and TVS content in household organic fractions, which range between 55%-80 % and 75%–90 %, respectively [4,12,13]. The higher MC can facilitate a faster decomposition rate of organic fractions via microbial activity. The higher TVS content indicates a more significant potential for organic matter conversion into valuable compost, enhancing nutrient-rich soil conditioning. Therefore, the combination of BOW and GW signifies potential feedstock for composting. Besides this, the pH values of 5.6 and 5.3 lie within an optimal range for composting, as they fall slightly on the acidic side.

**Table 4:** Physical-chemical characteristics of BOW and GW.

Parameters	Organic fraction	
	Biodegradable organic waste (BOW)	Garden waste (GW)
Bulk density (kg/m^3^)	513 (19.43)	59 (5.03)
Moisture content (%)	79.5 (1.01)	19.2 (0.36)
Total organic matter (%)	90.7 (0.39)	81.1 (0.73)
pH	5.6 (0.06)	5.3 (0.06)
Electrical conductivity (dS/m)	2.95 (0.21)	1.4 (0.08)
C/N ratio	18.36	26.04

Similarly, the EC of 2.95 and 1.4 dS/m is within an acceptable range, and it signifies the presence of soluble salts in the compost. Studies have suggested excessive salt concentrations can impede microbial activity and hinder the breakdown of organic matter [4,39]. The C/N ratio of 18.36 and 26.4 for BOW and GW, respectively, indicates a balanced C/N ratio essential for the composting process. Generally, a typical range of C/N ratio of 15–30 has been reported as the most favorable substrate for composting. Besides this, the composting process can proceed effectively without the risk of excessive nitrogen loss, ensuring the production of high-quality compost.

### 3.2 Batch-study: variation in physical-chemical parameters

#### 3.2.1 Temperature

**Fig. 2:**
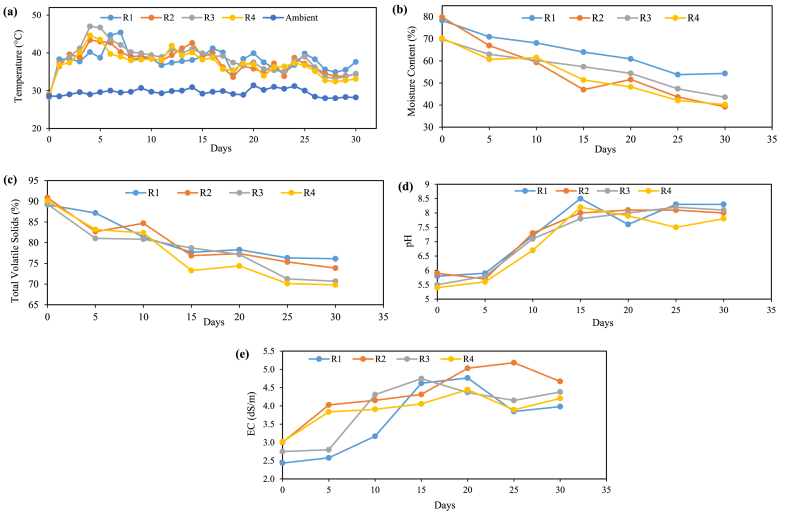
Schematic profile and trend analysis of composting process from a batch-scale study using reactors R1, R2, R3, and R4: (a) temperature, (b) moisture content, (c) total volatile solids, (d) pH, and (e) EC.

During the initial lag phase (0–10 days), the temperature fluctuation inside each reactor was found to be maximum, possibly due to microbial decomposition activities. Fig. 2 illustrates the daily temperature variation in four reactors. In Reactor R1, the reported temperature was around 40 °C for the first four days, as shown in Fig. 2 (a). However, the peak of 45.4 °C was reported on the 7th day. Generally, the typical temperature range from the 5th-15th days was 37–39 °C. However, there was a slight increase in temperature profile to 41.2 °C on the 16th day. At the same time, there was a slight decline in temperature on the 16th day but prevailed under the range of 35–40 °C until the 30th day. In Reactor R2, the highest temperature profile was reported as 42.7 °C on the 4th day, and after that, the temperature dropped slightly to 38 °C. The temperature again rose to 42.6 °C on the 14th day. In Reactor R3, the peak temperature was reported as 47 °C on the 4th day. However, the temperature remains within the 38–40 °C range until the 13th day. In Reactor R4, a peak temperature of 44.6 °C was detected on day 4, and it remained around 40 °C up to day 16, then slightly decreased. The statistical analysis results also showed minimum differences in temperature profiles among the four reactors during the composting period (P > 0.05).

In reactors, R2, R3, and R4, the highest temperature above the thermophilic range, i.e., ≥45 °C, was only reported between the 3rd to 7th days. This is likely due to the initial burst of microbial activities during the earlier phase. As organic matter decomposes, the microbial population rapidly multiplies, increasing metabolic heat production and elevated temperatures. This initial phase of high-temperature activity is a characteristic feature of the thermophilic stage in composting, which is driven by heat-loving microorganisms, such as thermophilic bacteria and actinomycetes, breaking down complex organic compounds. At the same time, the temperature remained <45 °C in the later phases of composting. This was likely due to the depletion of readily available organic fractions and the gradual transition from the thermophilic to the mesophilic stage. As the easily decomposable organic fractions were consumed, the microbial activities decreased. In the mesophilic phase, microorganisms that thrive at lower temperatures dominate the process, resulting in temperatures below the thermophilic range. A research also observed similar temperatures in the mesophilic range during their experiments on in-vessel composting of household waste [25]. The temperature remained slightly lower in reactors R2 and R4 than in reactors R1 and R3, mainly due to the turning frequency. Therefore, it was conclusive that the turning frequency allows better aeration and degradation of organic fractions.

#### 3.2.2 Moisture content

The initial and final MC in R1, R2, R3, and R4 was found to be 78.34 %, 79.84 %, 69.75 %, 70.25 %, and 54.32 %, 39.24 %, 43.58 %, 40.21 %, respectively, as shown in Fig. 2(b). The variations in the percentage reduction of MC observed in the batch-scale reactors (R1, R2, R3, R4) can be primarily attributed to differences in their operational conditions. Reactors R2 and R4 achieved higher MC reduction rates due to the inclusion of a turning process, which enhances aeration and microbial activity. This process facilitates more efficient moisture reduction through increased oxygen availability, stimulating aerobic microbial degradation. In contrast, R1 and R3 did not include turning, which likely hindered moisture loss by limiting aeration and reducing the overall efficiency of microbial activity.

Moreover, adding bulking agents in R3 and R4 increased porosity and improved airflow within the compost matrix. The bulking agents helped maintain a balanced moisture level, promoting evaporation and moisture reduction. In the absence of these bulking agents, R1 and R2 exhibited lower porosity, leading to reduced airflow and, consequently, lower moisture reduction rates. Furthermore, the limited moisture loss in R1 and R3 could be related to the formation of compacted zones within the compost material, which restricts gas exchange and moisture movement. The lack of turning in these reactors likely exacerbated compaction, further impeding moisture evaporation. On the other hand, the periodic turning in R2 and R4 helped to break up compacted areas, enhancing overall moisture loss.

#### 3.2.3 Total volatile solids

The TVS content is a critical parameter in assessing compost quality and reflects the organic content in the composting process. A decline in TVS is a direct indicator of the decomposition of organic materials into stable humus-like compounds. The efficient reduction of TVS is essential for high-quality compost, as it signifies a decrease in readily biodegradable organic matter, minimum odor issues, and leachate generation. The initial and final TVS content in R1, R2, R3, and R4 was found to be 89.17 %, 90.87 %, 89.31 %, 90.16 % and 76.12 %, 73.86 %, 70.68 %, 69.81 %, respectively, as included in Fig. 2(c) above. Reactors R2 and R4 exhibited a consistent and gradual decline in total volatile solids, suggesting effective organic matter decomposition. Turning in these reactors likely significantly promoted aeration and microbial activity, which are crucial for efficient composting.

In contrast, R1 and R3 displayed less consistent patterns, indicating less efficient decomposition. Reactor R1, with no turning provision and bulking agents, demonstrated a more erratic decline in TVS. At the same time, R3, comprising a bulking agent, resulted in a more stable TVS. The overall decreasing trend in all reactors underscores the ongoing organic matter decomposition during the composting process. It highlights the importance of operational conditions, such as turning and bulking agents, in influencing composting efficiency. It was found that the R2 and R4 had the most favorable results.

#### 3.2.4 pH and electric conductivity

In the present study, the initial pH for reactors (R1, R2, R3, R4) was found to be slightly acidic, which was in the range of 5.5–5.9. Fig. 2(d) presents the detailed variation in reactors' pH. The low pH often signifies the presence of organic acids synthesized during microbial growth. These acids temporarily lower the pH level, indicating the initial stages of decomposition. As the process advanced, a notable and consistent rise in pH was observed in all the reactors, which were near-neutral to mildly alkaline. This was likely due to the consumption of organic acids by a group of microbes prevailing under the thermophilic range. However, several studies have also claimed that this increase in pH aligns with the progression of the composting process towards its mature stages [26,27]. During the maturation phase, organic matter decomposition stabilizes, and organic acid production declines. Simultaneously, the microbial population transitions to favor species that decompose more complex organic compounds, such as lignin and cellulose. The decomposition of these compounds produces by-products like ammonia and other alkaline substances, which contribute to the observed increase in pH. The final pH range of 6–8 in the present study indicates a maturity stage of compost with improved stability.

Initially, the EC values were relatively low, which indicates low ion concentrations typical in the early composting phase, as shown in Fig. 2(e). As composting progresses, a consistent upward trend in EC was observed for all reactors. This trend demonstrates the ongoing transformation of organic materials into soluble forms, primarily ions. The increase in EC values signifies the decomposition of organic matter, releasing nutrients and salts into the compost matrix. In our study, the R1 and R4 were found to have more consistent increases in EC. This consistency suggests a steady breakdown of organic materials and a continuous release of soluble ions. The controlled conditions in these reactors, such as adequate aeration and optimal moisture levels, likely contributed to the stable increase in EC. These conditions support uniform microbial activity, leading to a gradual and predictable rise in ion concentrations.

In contrast, R2 and R3 revealed greater fluctuations in EC values, indicating dynamic changes in ion concentrations. These fluctuations can be attributed to variations in material composition, moisture content, and microbial community dynamics within each reactor. For instance, the periodic turning process in R2 could have introduced bursts of aeration, temporarily increasing microbial activity and ion release, followed by stabilization phases. Similarly, the lack of turning in R3 might have led to irregular aeration, causing intermittent surges and drops in microbial activity and corresponding EC values. The observed trends in EC also highlight the progressive ionic condition, which provides insights into the decomposition process and the changing solubility of organic matter. In R1 and R2, the increase in EC reflects the surge of soluble ions due to the reduced aeration process. The limited aeration rate could have led to anaerobic conditions, causing a rapid release of ions due to oxygen diffusion. Meanwhile, R3 and R4 displayed more controlled and consistent EC values, likely due to the more regular aeration and turning processes, which maintained aerobic conditions and steady microbial degradation.

### 3.3 Kinetic study from batch reactors

The kinetic study was conducted to assess the decomposition rate of the organic substrate during the composting process prevailing under different conditions. These studies offer essential information about the dynamics of the composting process, aiding in precisely adjusting key parameters and enabling accurate predictions of composting durations. The data set for organic degradation rate was analyzed using first-order kinetic ((1), (2)) and was compared between all the reactors as shown in Table 5 and Fig. S3. Reactor R4 reported the highest degradation rate, which signifies the fastest decomposition of organic matter among all reactors. This indicates that R4 is the most efficient in the breakdown of organic substrate. The reactor R2 has also reported an effective degradation rate of 0.0078 day^−1^. Therefore, it was conclusive that the reactors R2 and R4 reported the maximum degradation rate compared to R1 and R3. Similarly, two other studies on in-vessel composting using perforated vents reported degradation rates ranging from 0.0035 to 0.0067 day^−1^ and 0.0024 to 0.0059 day^−1^, respectively [31,40]. This also emphasizes the importance of aeration and mixing in accelerating organic matter decomposition.

**Table 5:** A kinetic study highlighting degradation rate and R^2^ values in reactors R1, R2, R3, and R4.

Reactors	R1	R2	R3	R4
Degradation rate (day^−1^)	0.0063	0.0083	0.0078	0.0098
R^2^	0.85	0.87	0.81	0.89

Adding bulking agents in R3 (0.0078 day^−1^) and R4 (0.0098 day^−1^) contributes to high degradation rates, underlining the value of creating a porous structure that enhances microbial activity. At the same time, Reactor R1 lacks turning and bulking agents, which results in a lower degradation rate (0.0063 day^−1^). In summary, the combination of turning, perforations, and bulking agents in R4 was most effective for promoting the rapid decomposition of organic fractions. At the same time, the relatively low R^2^ values (0.81–0.89) suggested that the model does not fully capture the complexity of the degradation process. Besides this, certain limitations and fitting performance of the first-order kinetic model are also influenced by different factors: (a) it oversimplifies the complex biological and chemical interactions, (b) it fails to account for the heterogeneity of organic waste and does not consider dynamic environmental fluctuations or microbial activity, and (c) also insufficient or variable experimental data can lead to lower R^2^ values, indicating poor fitting performance and less accurate predictions.

### 3.4 Pilot study: variation in physical-chemical parameters

#### 3.4.1 Temperature

**Fig. 3:**
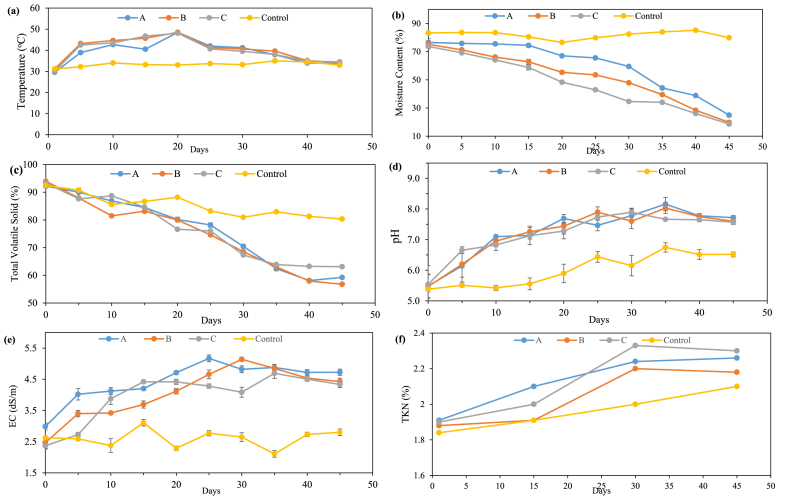
Schematic profile and trend analysis of composting process from the pilot-scale study using three-stage VDC for (a) temperature, (b) moisture content, (c) total volatile solids, (d) pH, (e) Electrical conductivity, and (f) total Kjeldahl nitrogen.

The average temperature in all three drums was found to be > 40 °C except control. The profile of the temperature variation in all the drums is presented in Fig. 3(a). The temperature profile in drum A was slightly lower than the other two drums (B and C) during the feeding phase. This was likely due to the higher MC in drum A during that phase. The peak temperature of 48.5 °C was observed in drum A on the 20th day. A similar trend of temperature profile was also observed in drums B and C with a peak of 48.5 °C and 48.1 °C, respectively. This was likely due to appropriate MC, higher bulking agent ratio, and aeration than in drum A. The overall temperature profile peaks in all the drums indicate an active decomposition of the organic substrate. During the transformation process, parameters like MC and TVS contents are expected to be in inverse proportion to the rise in the temperature of drums. After 30th days, the temperature profile began to decline in all three drums, which was appropriately close to the ambient temperature (35 °C).

In the thermophilic phase (≥45 °C), the temperature in all the drums was not retained as the MC was lower than the optimum required (45–65 %) in the drums. It was conclusive that the daily mixing through the mechanical system could not trap heat which was generated due to microbial activities. However, the daily mixing mechanism helped in the appropriate aeration process. It was also observed that the generated leachate was appropriately mixed during the initial process (1st-30th days). However, after the 30th day, the mixing process was found to have adverse effects like over-mixing and water clogging inside all the drums. The control drum's temperature remained almost below 40 °C throughout the experiment. This was likely due to the higher MC (around 80 %) and no aeration facilities from perforation vents. A similar trend was observed in statistical analysis, which showed significant differences in temperature variations among drums (A, B, C) during the composting period (P = 0.007). Therefore, it was observed that the MC and TVS content in the control drum was higher than all other drums.

#### 3.4.2 Moisture content

The moisture content of the residential BOW was generally higher (∼83 %). The initial MC in drums A, B, C, and control was 76.5 %, 75.2 %, 73.7 %, and 83.2 %, respectively as shown in Fig. 3(b). During the feeding phase, the MC was approximately 75 % in drum A. The MC in drums B and C was found to be 62.8 % and 58.8 %, respectively. The GW, which is comprised of major fractions of dried leaves, facilitated the reduction of MC in compost feed samples. It was also observed that leachate generation was much less in all the drums compared to the control. As the percentage of the addition of bulking agents increased, the leachate generation decreased. Due to the degradation process and turning effect, the particle size was reduced significantly, and the dried leaves helped maintain the feed's porosity [41]. The MC of drums B and C decreased sharply after 20 days, which can be because of more evaporation as both drums were placed at a height of almost 1m and 1.5 m above the base. The statistical analysis results also showed a significant difference in mean-variance (μ_difference_ = 23.54) between drum B (μ = 334) and C (μ = 357). This suggests a substantial difference in MC formation inside drums B and C. Besides this, the high evaporation rate can also be due to the overturning effect, resulting in reduced biological activity. It was also observed that the reduction of organic matter was low in Drum B compared to the early phase.

The MC in Drum C was found to be less on the 30th day, around 34.6 %. Therefore, approximately 600 mL of water was added to the drum to maintain the MC in Drum C. However, no significant change was observed in MC, possibly due to the higher ambient temperature and evaporation rate. The final MC of compost from drums A, B, C, and control was 24.9 %, 19.8 %, 18.6 %, and 79.9 %, respectively. The higher MC in the control was due to the accumulation of leachate generated during the process. Generally, the higher MC also hinders the overall biological process, resulting in a rise in temperature and evaporation losses. Besides this, the problems related to foul odor and flies were very high in control units, mainly due to the anaerobic conditions inside the composter. At the same time, there were no issues related to foul odor emission and breeding of flies in all three drums (A, B, and C) even after 25 days of functioning.

#### 3.4.3 Total volatile solid

The initial concentration of TVS was 92–93 % of total waste. The detailed trend analysis of TVS content in drums A, B, C, and control is presented in Fig. 3(c). At the end of the feeding phase, the percentage of TVS was almost the same in all four drums, around 84 %. The decomposition rate was faster in Drum B than in the other two drums. During the decomposition phase, the TVS was reduced significantly with time, resulting in heat generation and a rise in the temperature profile. In the decomposition phase, the reduction of TVS in drums A, B, and C were 25.2 %, 26.4 %, and 21.5 %, respectively. In Drum C, the percentage of dried leaves was higher as it primarily comprises lignocellulose biomass, which is less degradable. Therefore, Drum C has reported a minimum reduction in TVS content. Based on the statistical analysis, it was conclusive that there was a significant difference in TVS content for drum C (μ = 135) than A (μ = 165) and B (μ = 163). It suggests that drum C had a considerably low degradation rate. The TVS reduction was around 12 % in the control drum, and this can be due to the minimum aeration and higher MC.

#### 3.4.4 pH

Initially, the pH values for drums A, B, C, and control were 5.49, 5.47, 5.54, and 5.38, respectively. This initial low pH was likely due to the production of organic acids by microbial activities, which results in acidification. During the complete composting process, the average pH reported in drums A, B, C, and control was found to be 7.25, 7.22, 7.19, and 6.01, as shown in Fig. 3(d). The maximum pH values reported for drums A, B, and C and control were 8.16, 8.03, 7.89, and 6.75, correspondingly. It was conclusive that the high pH value in drums enhanced the reduction of organic acids and the production of alkaline compounds, resulting in a more alkaline pH. While the statistical analysis with p < 0.001 proved that there was a significant difference in pH value in all the drums, it was found that the peak value was reported between the 30th to 35th days of the composting process. This provides the optimum consumption of organic acids, leading to an increase in pH and compost maturity stage. It also shows that the expected reduction of nitrogen was higher in terms of ammonia gas. Therefore, the pH was again reduced to around neutral. The pH was above 7 during the degradation phase, which might be the reason for significantly less odor emission in drums A, B, and C. In the control drum, the pH remained in the acidic range of 5–6.5, resulting in higher odor emission.

#### 3.4.5 Electrical conductivity

Initially, the EC in all the drums was in the range of 2.5–3 dS/m, as shown in Fig. 3(e). The EC indicates the feedstock's overall salinity and reflects the compost's quality. The higher value of EC shows that it could lead to potential phytotoxicity in the compost. The EC was found to be at a lower side in Drum C, possibly due to the higher bulking agent ratio. Finally, the EC in drums A, B, and C was 4.72, 4.42, and 4.33 dS/m, respectively. As per the Solid Waste Management (SWM) Rules, 2016 [India], it was found that the permissible standard for EC in all the drums was on the higher side, i.e., >4 dS/m [42]. However, these higher values can be reduced with the increase in retention duration of the compost in drums. The higher values of EC could be likely due to the less soluble ions drained out through leachates. Besides this, the higher values slightly affect the germination of the seed. Therefore, it is recommended to use compost after mixing it with the soil with slightly lower EC; this might neutralize the overall EC of compost. The EC for control was found to be within the range of 2.64–3.1. However, no definite trend was observed in the control drum as there was a higher variation in EC value.

#### 3.4.6 Carbon/nitrogen ratio

Generally, an ideal C/N ratio indicates a balanced environment for microbes, ensuring efficient decomposition of organic substrate and producing high-quality compost. The reduced C/N ratio supports the breakdown of carbon-rich organic fractions via microbes [4,12]. This process causes a release of carbon dioxide and nitrogen-enriched compounds in the form of matured compost residues [4]. In this study, the final C/N ratio of compost residues in drums A, B, C, and control was found to be 14.9, 15, 12.1, and 17.3, in that order. As per the SWM Rules, 2016, the C/N ratio of the final product must be less than 20 [42]. Therefore, it is conclusive that the final C/N ratio in drums A, B, and C was also under the permissible limit. The percentage reduction in the C/N ratio in drums A, B, C, and control was found to be 40.5 %, 40 %, 51.6 %, and 30.8 %, respectively. It was also learned that the drums had a higher performance efficiency and higher C/N reduction compared to the control.

#### 3.4.7 Total Kjeldahl nitrogen

TKN is a crucial indicator of nitrogen content and decomposition in compost. The increasing TKN values in all drums indicate a gradual accumulation of nitrogen, primarily due to the breakdown of organic matter. Drums A, B, and C exhibit consistent increases in TKN, with Drum C reaching the highest value of 2.33 as shown in Fig. 3(f). This suggests that adding bulking agents in Drum C may have facilitated nitrogen retention and promoted organic matter decomposition. In the initial stages of composting, organic materials include nitrogen-rich compounds which are broken down by microbes. As the composting process advances, microbes metabolize organic matter, which also releases nitrogen in various forms [43,44]. This nitrogen becomes more concentrated as the carbon-rich organic matter is consumed, leading to higher TKN values. The rising TKN levels support the gradual transformation of complex organic compounds into simpler, nitrogen-rich compounds, which are essential for plant nutrition and contribute to the nutrient content of the mature compost [45,46]. This trend also reflects the progressive maturation of compost as it evolves from raw organic material to a valuable soil conditioner and fertilizer [28]. In contrast, the control drum had a slower rate of TKN increase. This indicates that the absence of specific composting aids can result in less efficient nitrogen accumulation [45].

### 3.5 Maturity test

**Fig. 4:**
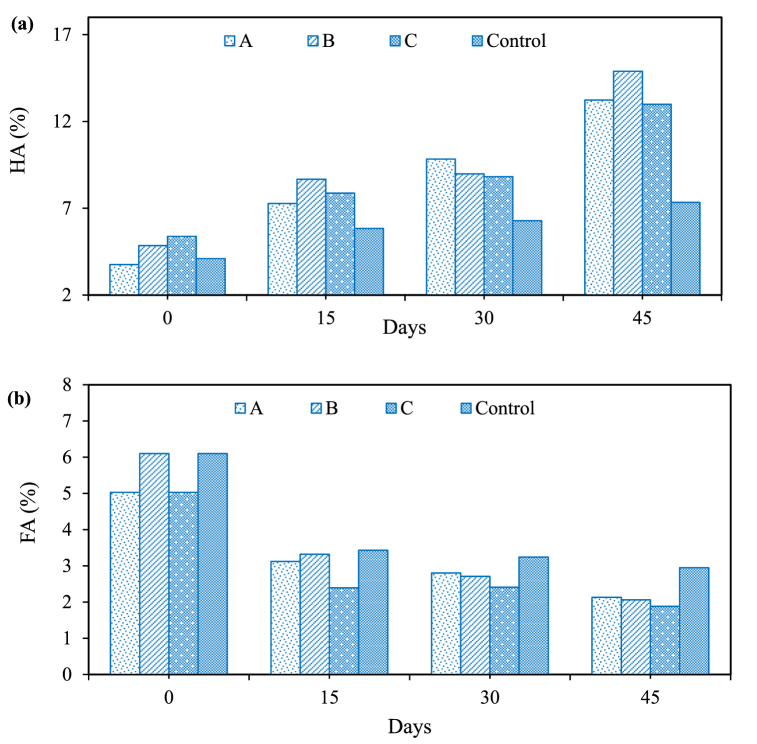
Percentage variation in: (a) humic and (b) fulvic acid content in compost samples during the composting process in three-stage vertical drum composter.

HA and FA are organic substances that enhance soil structure, improve nutrient retention, and promote plant growth. The ranges of HA and FA content in drums A, B, C, and control were 3.75%–13.23 %, 4.85%–14.89 %, 5.38%–13 %, and 4.1%–7.34 %; and 5.03%–2.13 %, 6.1%–2.06 %, 5.03%–1.88 %, and 6.1%–2.95 %, respectively as shown in Fig. 4(a & b). The increasing and declining trend in HA and FA content was likely due to the transformation of organic fractions into a mature state of compost. A similar trend was found to be reported by other studies [47,48] as compost matures, the complex organic fractions are transformed into more stable forms such as HA, which results in higher HA content. Conversely, the breakdown of soluble organic compounds leads to reduced FA content, representing a shift towards more stable mature compost [47,48]. Besides this, the microbial community utilizes FA for its metabolic growth rate, which causes the organic substrate to transform into HA. Therefore, the HA content was found to be higher than FA. At the same time, the control drum exhibited lower HA and FA content as it lacks specific composting aids and conditions that promote humification [47]. The absence of bulking agents and controlled conditions in the control drum results in less efficient humic substance formation and preservation.

**Fig. 5:**
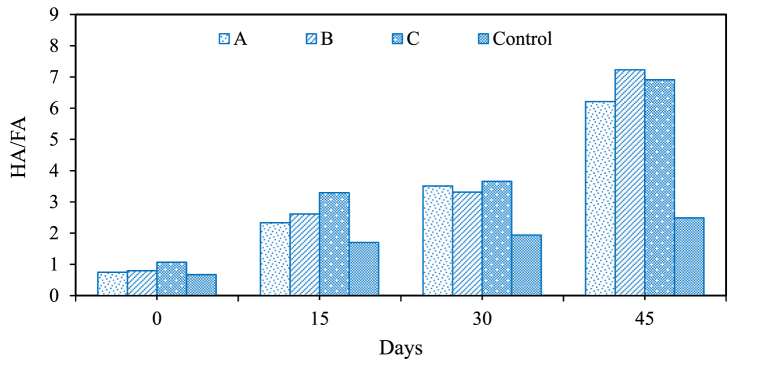
Percentage variation in humic to fulvic acid (HA/FA) ratio in three-stage vertical drum composter.

The HA/FA ratio in compost maturity indicates the degree of humification. This suggests advanced compost maturity, increased stability, and improved soil-conditioning properties, as humic substances enhance soil structure and nutrient retention. In this study, the HA/FA ratio in drums A, B, C, and control was 0.74–6.21, 0.79 to 7.22, 1.06 to 6.91, and 0.67 to 2.48, respectively, as shown in Fig. 5. The increasing trend of HA/FA ratio indicates the maturation of compost as complex organic compounds transform into stable humic substances over time. A research suggested that the standard HA/FA ratio for a mature compost typically falls in the range of 5–15 [49]. This indicates well-humified compost with improved soil-conditioning and nutrient-retention properties exist [37]. Therefore, it is conclusive that the compost prepared using drums has been under the permissible limit. The lower HA/FA ratio in control indicates limited humification due to unregulated composting conditions, resulting in a less mature compost with lower levels of humic substances [31,50,51].

### 3.6 Germination index

**Fig. 6:**
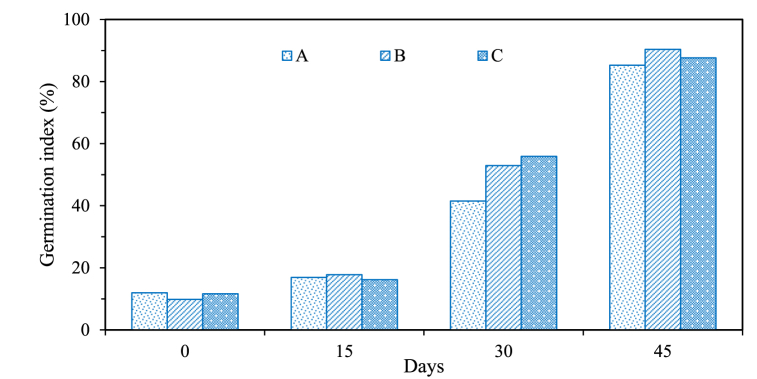
Percentage variation in germination index in three stage vertical drum composter.

In this study, the germination test was performed to assess the phytotoxicity of the final compost product. The test signifies the maturity stage of compost, suitable for enhancing soil quality and promoting plant growth. According to a study, compost with a GI exceeding 80 % is classified as mature, while those with a GI surpassing 90 % are considered highly mature [30]. In our study, the initial GI value in drums A, B, and C was 12 %, 9.8 %, and 11.6 %, respectively. The lower initial GI values are attributed to the presence of less mature or decomposed organic materials at the beginning of the composting process [52,53] as shown in Fig. 6. This incremental trend in GI values indicates the increasing incorporation of fresh organic matter into the compost, which adds more nitrogen-rich components [29,38]. This gradual infusion of organic fractions results in a higher GI, signifying organic matter's ongoing decomposition and transformation in the compost. Finally, the GI in drums A, B, and C was 85.3 %, 90.4 %, and 87.6 %, respectively. The higher GI values indicate improved phytotoxicity reduction during composting [29]. Besides this, it also suggests that the compost is less harmful to plant germination, indicating a decrease in potentially toxic substances and enhanced compost quality.

### 3.7 Quality assessment of compost samples

**Fig. 7:**
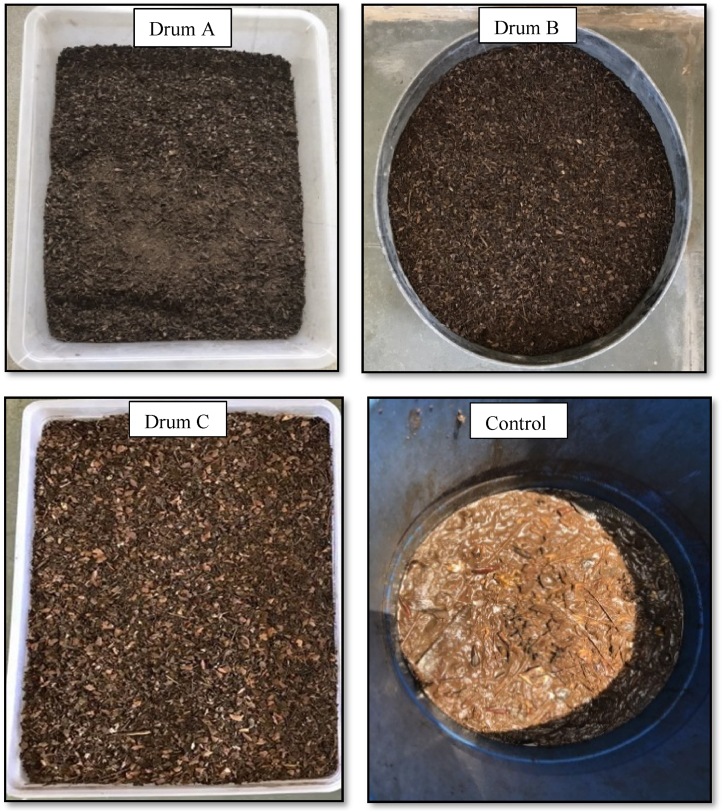
Final quality of compost procured from drums A, B, C, and control after 45 days.

The quality assessment of compost samples from drums A, B, C, and control was compared in alignment with the SWM Rules, 2016 through a range of parameters [42]. The compost samples were collected and analyzed from each drum after 45 days. The final physical-chemical characteristic of compost samples has been discussed in Table 6. Fig. S4 represents the aging and transformation process of BOW into nutrient-rich compost samples on the 1st, 15th, 30th, and 45th day in three-stage VDC. Fig. 7 has the final quality of compost procured from drums A, B, C, and control.

**Table 6:** Physical-chemical characteristics of final compost samples and their comparison with standard values as per SWM Rules, 2016 [42].

Parameters	A	B	C	Control	SWM rules, 2016
Bulk density (g/cm^3^)	0.31	0.28	0.24	–	<1
MC (%)	24.92	19.8	18.63	79.91	15–20
TVS (%)	59.25	56.79	63.13	80.34	–
pH	7.72	7.6	7.57	6.52	6.5–7.5
EC (dS/m)	4.73	4.43	4.34	2.80	≤4
C/N ratio	14.85	15.03	12.08	14.30	≤20
TOC (%)	32.92	31.55	35.07	44.63	Minimum 12
TKN (%)	2.26	2.18	2.3	2.01	–
HA (%)	13.29	14.89	13.0	7.65	–
FA (%)	2.13	2.06	1.88	2.95	–
Weight reduction (%)	85.3	80.9	78.8	–	–
Volume reduction (%)	75.5	65.1	54.7	–	–
Yield (%) (<4 mm)	13.0	15.8	15.9	–	–
Colour	Black	Black	Dark Brown	–	Dark brown/black
Cu (mg/kg)	95	110.3	172.2	105.5	300 (max.)
Ni (mg/kg)	14.5	8.5	18.4	11.9	50 (max.)
Pb (mg/kg)	38.5	47.6	41.6	46.4	100 (max.)
Zn (mg/kg)	282.5	295	382.5	262.5	1000 (max.)

#### 3.7.1 Physical characteristics

The bulk density is an indicator of compost weight and structure. The bulk density of compost from drums A, B, and C was 0.31 g/cm^3^, 0.28 g/cm^3^, and 0.24 g/cm^3^, respectively. These values are lower than the recommended threshold of <1 g/cm^3^ as per SWM Rules, 2016 [42]. This indicates that the compost from reactors A, B, and C has a desirable low bulk density, suggesting good porosity and soil aeration potential.

#### 3.7.2 Moisture content

The drums A, B, and C exhibit MC values of 24.92 %, 19.8 %, and 18.63 %, respectively. While these values are higher than the suggested range of 15–20 % [42], they are still within an acceptable range for compost quality.

#### 3.7.3 Chemical properties

The compost pH values for drums A, B, and C were 7.72, 7.6, and 7.57, respectively. The pH numbers for the compost samples were within the acceptable range of 6.5–7.5 [42]. The EC values for reactors A, B, and C were 4.73, 4.43, and 4.34 dS/m. The value was also below the recommended threshold of ≤4 dS/m. The C/N ratios for reactors A, B, and C were 14.85, 15.03, and 12.08, in that order. The values for the C/N ratio meet the criteria of ≤20. These results collectively indicate that the compost from reactors A, B, and C aligns well with the SWM Rules, 2016 for pH, EC, and C/N ratio [42].

#### 3.7.4 Organic content

The total organic carbon (TOC) percentages for reactors A, B, and C were 32.92 %, 31.55 %, and 35.07 %, respectively. These values exceed the minimum requirement of 12 % set by SWM Rules, 2016 [42]. This also indicates the presence of higher lignocellulose biomass content in organic fractions used in the compost. It is recommended to adjust the percentage of bulking agents in the composting process as per the working duration of the composter. Also, the TKN percentages for reactors A, B, and C were 2.26 %, 2.18 %, and 2.3 %, respectively. It was conclusive that the existing compost sample meets the general criteria for nitrogen content.

#### 3.7.5 Humic and fulvic acids

The final HA and FA values in drums A, B, and C were found to be 13.29 %, 14.89 %, and 13 %; and 2.13 %, 2.06 %, and 1.88 %, respectively. These numbers suggest the presence of substantial humic substances in the compost, which enhances its soil-conditioning properties.

#### 3.7.6 Weight and volume reduction

The weight and volume reduction for reactors A, B, and C were 85.3, 80.9, and 78.8; and 75.5, 65.1, and 54.7, respectively. The overall weight and volume reduction were significantly better, indicating the organic substrates' efficient decomposition inside the composter. However, no standard permissible limit for weight and volume reduction has been prescribed by the SWM Rules, 2016 [42].

#### 3.7.7 Yield and color

The yields of <4 mm particles for reactors A, B, and C were 13 %, 15.8 %, and 15.9 %, respectively, thus indicating the size distribution of the compost. The color of the compost from reactors A, B, and C was black, suggesting the compost samples' maturity state. The color of the control was dark brown, which shows a poor decomposition rate inside the composter.

#### 3.7.8 Heavy metal concentration

The concentrations of heavy metals, including copper (Cu), nickel (Ni), lead (Pb), and zinc (Zn) in compost from reactors A, B, and C were found to be within the permissible limits [42], demonstrating the absence of contamination. In conclusion, the compost from reactors A, B, and C exhibits favorable physical and chemical characteristics aligning well with the SWM Rules, 2016 [42] and demonstrating its suitability for use as a soil conditioner and fertilizer with the added benefit of low heavy metal content, hence, making it an environmentally safe product.

## 4 Conclusions

This study comprehensively assessed on-site composting using a three-stage vertical drum composter for household biodegradable organic waste. The aim was to investigate the impact of factors such as aeration, turning mechanisms, bulking agents, and process parameters on compost quality. It also aspires to provide a robust framework for process optimization and improved compost quality. The key findings from the present study are summarized below.

i) The provision for aeration, facilitated by perforated vents and regular turning mechanisms, significantly influenced the quality of the final compost.
ii) Moisture content, turning frequency, and the addition of bulking agents emerged as critical determinants in the composting process, affecting heat generation and degradation process.
iii) The maturity of the compost, assessed through HA/FA ratio (7–7.5) and GI (>85 %), suggested its nutrient-rich and soil-conditioning properties. Therefore, it was conclusive that compost samples were suitable for vegetation and plantation activities.
iv) Over the 45-day composting period, a notable reduction in moisture content was achieved (∼20 %) with temperatures ranging <50 °C due to daily mechanical turning.
v) Based on the kinetic study, it was conclusive that the addition of bulking agents in the reactor (0.0078–0.0098 day^−1^) contributed to high degradation rates, underlining the value of creating a porous structure that enhances microbial activity.

## Ethical statements

All subjects gave their informed consent for inclusion before they participated in the study.

## Funding

The authors declare that no external funds or grants were received for this study.

## CRediT authorship contribution statement

**Dakshesh Chimanbhai Saypariya:** Writing – original draft, Software, Methodology, Investigation, Formal analysis, Data curation. **Deval Singh:** Writing – review & editing, Writing – original draft, Validation, Software, Methodology. **Anil Kumar Dikshit:** Supervision, Conceptualization. **Mohan B. Dangi:** Writing – review & editing, Supervision, Resources, Conceptualization.

## Declaration of competing interest

The authors declare that they have no known competing financial interests or personal relationships that could have appeared to influence the work reported in this paper.
