# Supplementary material for: Composting of organic fraction of municipal solid waste in a three-stage biodegradable composter
Source: Heliyon. 2024 Sep 4;10(17):e37444. doi: 10.1016/j.heliyon.2024.e37444 (PMC11408821; doi:10.1016/j.heliyon.2024.e37444)
Supplement: Multimedia component 1 [file mmc1.docx]

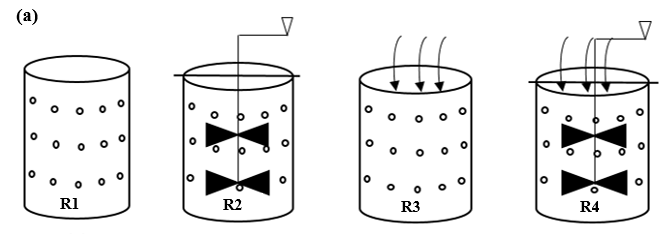


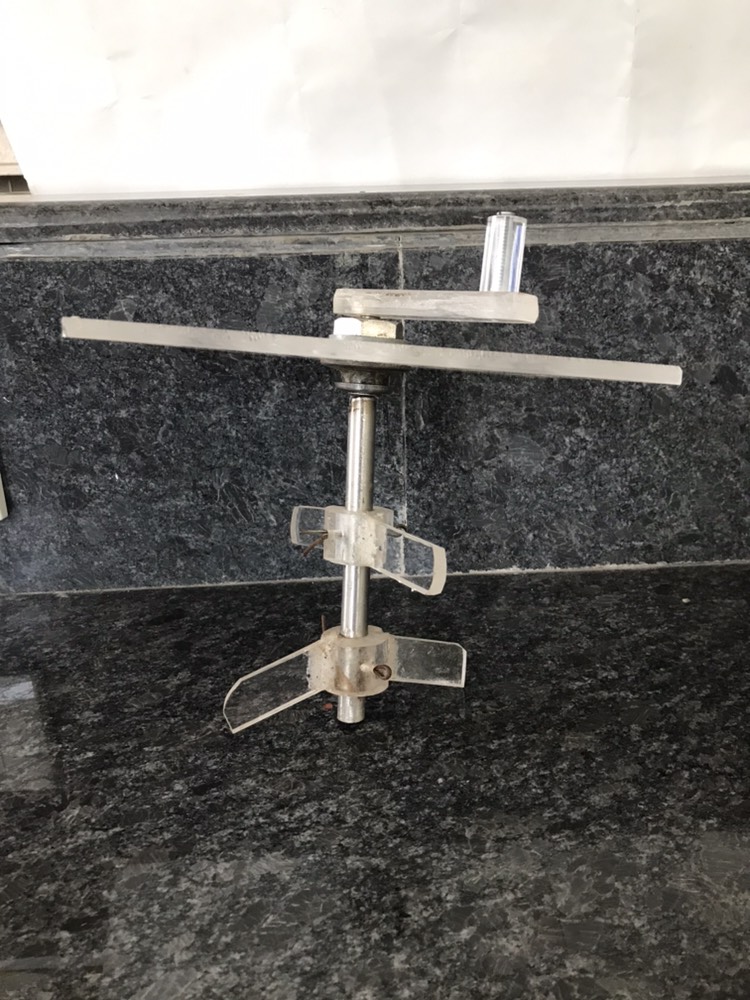

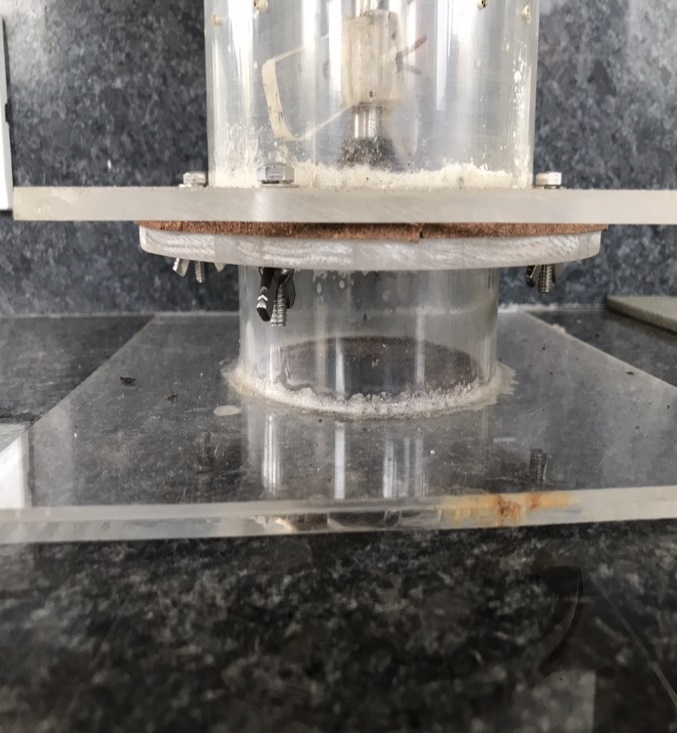


**(b)**

**(c)**

Fig. S1. Schematics of – (a) lab-scale reactors, (b) turning mechanism, and (c) leachate collection base in reactors.


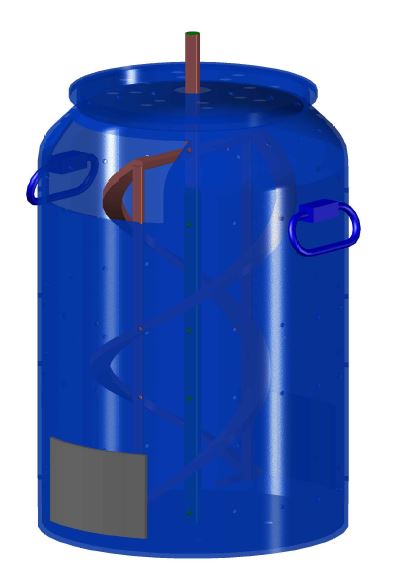

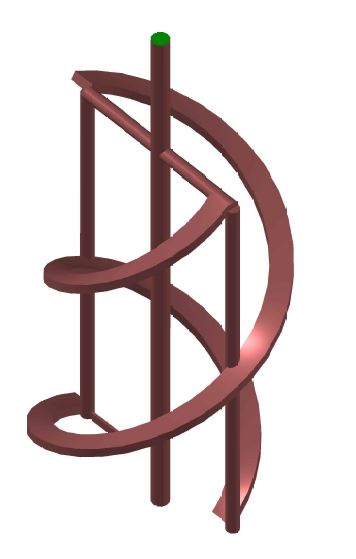

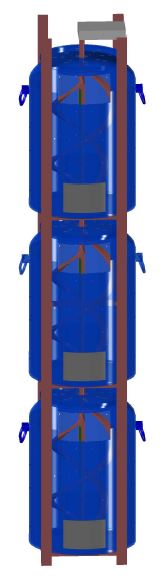


**C**

**B**

**A**


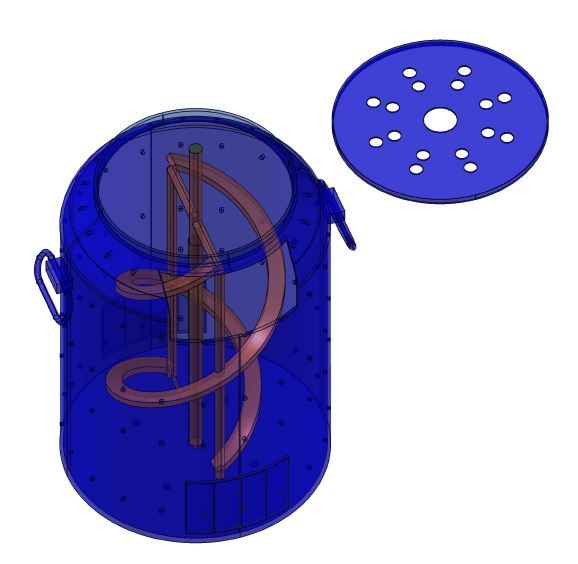


Fig. S2. 3D design model of three-stage vertical drum composter.

Fig. S3 Curve fitting of the first-order kinetic equation in – (a) R1, (b) R2, (c) R3, and (d) R4.


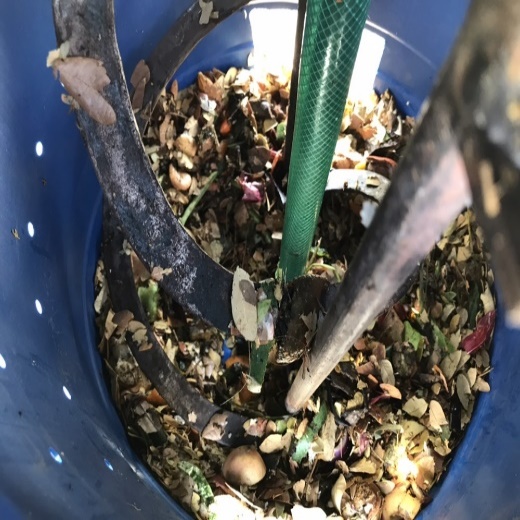

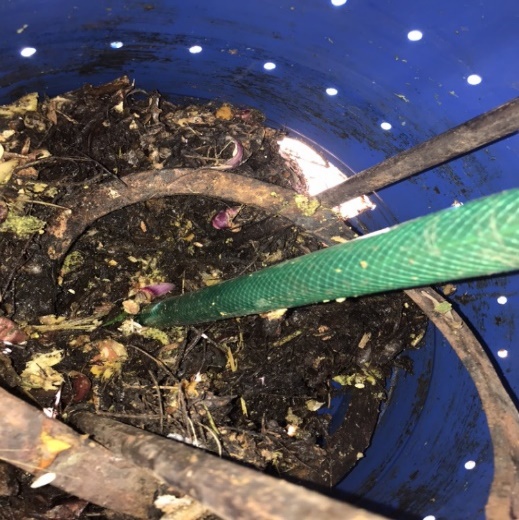


Day 1 Day 15


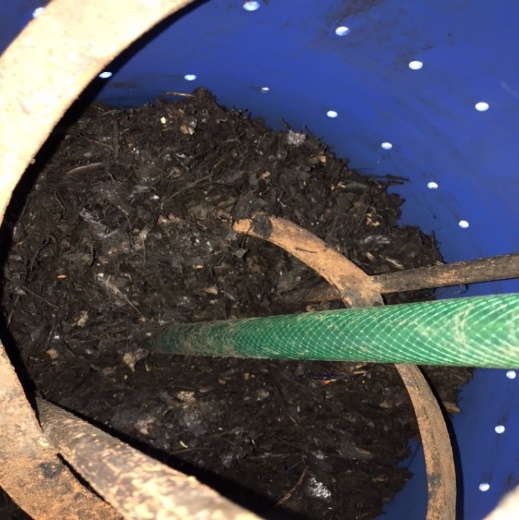

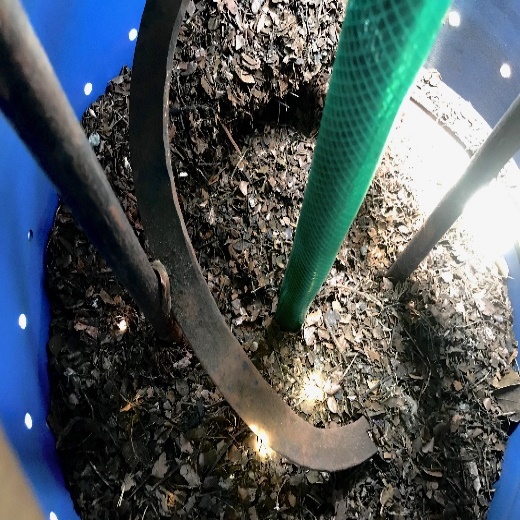


Day 30 Day 45

Fig. S4. Aging and transformation process of BOW into nutrient-rich compost samples on 1^st^, 15^th^, 30^th,^ and 45^th^ day in three-stage VDC.
